# The Essential Role of Medical Monitors in Clinical Trials

**DOI:** 10.7759/cureus.92388

**Published:** 2025-09-15

**Authors:** Gerald L Klein, Elizabeth Barabash, Roger E Morgan, Gail Brown, Mark Tulchinskiy, Freddy Byrth, Emilia Jones Amaowei, Pavle Vukojevic, Shabnam Vaezzadeh, Katie-Louise Dawson, Gabriel Cohn, Stephen Haworth, Anne Blackwood-Chirchir, Johannes Wolff, Angela Overton

**Affiliations:** 1 Medical Affairs, MedSurgPI, Pittsboro, USA; 2 Pharmacology and Toxicology, East Carolina University Brody School of Medicine, Greenville, USA; 3 Strategy and Development, People Value Research Ltd., Ipswich, GBR; 4 Clinical Development, MedSurgPI, Raleigh, USA; 5 Internal Medicine and Medical Oncology, MedSurgPI, San Jose, USA; 6 Medical Affairs, MedSurgPI, Morrisville, USA; 7 Clinical Operations, MedSurgPI, Chapel Hill, USA; 8 Medical Affairs, MedSurgPI, Pearland, USA; 9 Rheumatology, Optimapharm, Belgrade, SRB; 10 Medical Affairs, Exquisite Biomedical Consulting, Vancouver, CAN; 11 Pharmaceutical Medicine, MedSurgPI, Chalfont, USA; 12 Obstetrics and Gynecology, MedSurgPI, Longmeadow, USA; 13 Internal Medicine, MedSurgPI, London, GBR; 14 Oncology, MedSurgPI, El Cerrito, USA; 15 Oncology, MedSurgPI, Puyallup, USA; 16 Pharmacovigilance, Prevail InfoWorks Inc., Philadelphia, USA

**Keywords:** clinical trials in all phases (i-iv), drug and device, investigator brochure, medical monitor, protocols, safety patient, us fda

## Abstract

Medical monitors (MMs) play a critical and often underrecognized role in ensuring patient safety, regulatory compliance, and scientific integrity during clinical trials. Particularly in early-stage pharma, biotechnology, and medical device development, the MM serves as an independent safeguard against bias and unanticipated risks. This editorial outlines the rationale, responsibilities, and operational value of the MM role, emphasizing its function in document review, real-time safety oversight, and regulatory alignment. Through illustrative case studies and current best practices, we argue that the independence and objectivity of the MM is a strategic business necessity, not merely a regulatory checkbox.

## Editorial

Introduction and background

In the rapidly evolving landscape of medical technology, pharma, and biotechnology innovation, clinical trials must balance speed of execution with rigor, safety, and ethical integrity. Emerging companies frequently operate under limited resources and compressed timelines, which may lead them to ignore the critical functions of the Medical Monitor (MM). The MM acts as a dedicated physician and clinical scientist, independent of the study sponsor, to oversee participant safety and trial integrity across all phases of a clinical program [1]. The MM should be trained in Good Clinical Practice (GCP), drug safety, and FDA regulations, along with training and/or experience in the therapeutic area under study. Failure to implement robust and unbiased medical monitoring can result in delayed trial timelines, compromised patient safety, regulatory censure, and a loss of public trust, all of which significantly outweigh the cost of proper oversight [2]. These responsibilities include drafting or reviewing key study documents. These include the protocol, investigator's brochure, medical monitoring and safety plan, Data and Safety Monitoring Board (DSMB) or Safety Review Committee (SRC) charters, responding to site inquiries, training clinical staff (on the study and relevant safety regulation), and collaborating with pharmacovigilance and regulatory teams.

Key document review

Protocol Review and Oversight

The MM evaluates clinical trial protocols to ensure a justifiable risk-benefit profile, scientific validity, and operational feasibility [3]. This includes confirming adverse event (AE) definitions and reporting, minimizing unnecessary procedures, and integrating robust safety monitoring. The MM review should include several critical elements.

The MM evaluates whether the anticipated therapeutic benefit justifies the potential risks, integrating available preclinical and clinical evidence with the study’s design, dose rationale, and prespecified safety monitoring parameters [4]. It ensures the study is designed to answer meaningful clinical questions in relation to the study objective. This includes confirming that endpoints are clinically appropriate, inclusion/exclusion criteria are appropriate, and the statistical analysis plan aligns with the study objectives [5]. The monitor confirms clear definitions for AEs, serious AEs (SAEs), and other key safety endpoints such as dose-limiting toxicities (DLTs) or adverse events of special interest (AESIs). The MM ensures these are aligned with regulatory guidance (e.g., ICH E2A, FDA, and other international regulatory bodies) [6,7].

In addition, the MM evaluates the procedural burden and patient-centricity of the protocol, such as identifying and minimizing unnecessary or duplicative assessments (e.g., redundant lab work or imaging) that do not enhance safety or efficacy evaluation, thereby improving subject retention and enrollment feasibility [8]. The MM also ensures that a comprehensive and integrated safety monitoring plan is embedded in the protocol. This includes predefined thresholds for dose interruptions, patient and study stopping rules, unblinding procedures, or DSMB engagement if applicable [9,10].

Finally, the MM reviews the operational feasibility of the protocol across intended clinical trial sites. This assessment considers the availability of the target patient population, the complexity and frequency of study visits and procedures, and the logistical challenges associated with investigational product administration, handling, and storage. The goal is to ensure that the protocol is not only scientifically rigorous but also realistically executable in diverse settings. A well-known failure in protocol oversight occurred during the TGN1412 trial conducted in 2006 [11-13]. In this first-in-human study, six healthy volunteers experienced cytokine storm and multi-organ failure following the administration of a CD28 superagonist monoclonal antibody [11]. The protocol failed to anticipate human immune responses. This unfortunate event prompted major reforms in Phase 1 safety practices, including enhanced medical monitoring mandates [12].

Investigator’s Brochure (IB)

The MM ensures that the IB comprehensively presents preclinical and clinical data, translating pharmacologic properties into actionable safety information for investigators [7]. This includes clarifying potential risks, contraindications, AEs, SAEs, and AESIs that may emerge during human exposure. It is required to keep the IB updated appropriately.

Medical Monitoring Plan (MMP)

The MMP delineates the MM’s responsibilities and formalizes the communication pathways required for effective safety oversight [9]. Table 1 illustrates how the MMP operationalizes the MM's responsibilities through defined communication channels to ensure consistent and timely safety oversight [14]. The plan should clearly outline both mandatory and optional responsibilities; this distinction ensures transparency in role expectations, facilitates consistent execution across study sites, and supports compliance with regulatory and GCP requirements.

**Table 1:** Key components of the MMP MMP: Medical monitoring plan, SAE: Serious adverse event, SUSAR: Suspected unexpected serious adverse reactions, UADE: Unanticipated adverse device effects

Responsibilities	Description
SAE review	Review of SAEs within 24 hours and work with the pharmacovigilance team to ensure a timely report to the FDA or equivalent if not in the USA.
SUSAR/UADE assessment	Evaluation of suspected unexpected serious adverse reactions or unanticipated adverse device effects as applicable, in accordance with regulatory requirements for medical device studies.
Site support	Ongoing support to investigational sites regarding causality assessment and adverse event grading.
Safety and compliance review	Review of safety data, protocol deviations, and subject eligibility criteria.
Committee participation	Leadership or participation in committees such as the Safety Monitoring Committee and attendance at scheduled investigator meetings.

MM-Specific Duties

Table 2 summarizes the core duties and optional contributions of the MM in clinical trials, distinguishing the essential, non-delegable safety oversight functions from the value-added activities that can further enhance trial quality, operational efficiency, and stakeholder engagement.

**Table 2:** The MM-specific duties MM: Medical monitor, IB: Investigator's brochure, AE: Adverse event, SAE: Serious adverse event, AESI: Adverse event of special interest, DSMB: Data and Safety Monitoring Board, MeDRA: Medical Dictionary for Regulatory Activities, WHO: World Health Organization, SIV: Site initiation visit, SRC:  Safety Review Committee

Core duties	Optional duties
Review and comment on the IB and protocol	Review coding (MeDRA and WHO)
Write the medical monitoring plan	Educate both the site and sponsor teams on trial-related medical and safety issues
Review and approve a safety management plan	Support site selection or medical feasibility assessments
Review relevant protocol deviations for participant safety and data integrity	Present safety content at the investigator meetings, or at the SIV meeting and sponsor meetings
Review medical history, concomitant medications, vital signs, laboratory data, and physical exams	If the protocol stipulates, the MM should approve participant eligibility (typically seen in complex oncology studies)
Offer prompt and readily accessible medical support (typically available 24/7 to clinical sites for all protocol-related inquiries, including AEs and investigational product questions)	Review and comment on the DSMB/SRC charter and assist with proper documentation as required
Recommendations on continued participation for high-risk individuals [15]	
Review SAEs, AEs, and AESIs (causality and assessment of AEs and SAEs) [16]	
Escalate potential safety signals or trends to the appropriate governing body, such as the sponsor or an oversight committee (e.g., DSMB)	
Manage relationships with clinical investigators and their site staff	

Safety Concerns and Escalation

The MM is tasked with identifying and escalating emerging safety concerns. This may lead to assisting with regulatory interactions, which include trends in AE events, unexpected toxicities, or deviations that may threaten participant welfare or data validity [17]. The MM may recognize a safety signal and recommend halting enrollment, modifying protocol elements, or convening the DSMB [17]. The MM may support the pharmacovigilance team in identifying safety signals, conducting signal evaluation, investigating the mechanism of toxicity, assessing risk-enhancing variables, and determining appropriate risk mitigation strategies.

A pivotal example is the 2016 Juno Therapeutics JCAR015 trial, which was terminated following several patient deaths from cerebral edema in a chimeric antigen receptor T-cell (CAR-T) phase II trial for acute lymphoblastic leukemia [18]. Initial attribution to chemotherapy preconditioning was later questioned. This event underscored the need for signal detection and continuous, independent safety surveillance in high-risk studies involving novel modalities like cell or gene therapies [19].

Regulatory Alignment and Reporting

Regulatory authorities increasingly emphasize the need for qualified and independent medical oversight. The U.S. FDA, European Medicines Agency (EMA), and International Council for Harmonization (ICH) require prompt reporting of SUSARs and stress the value of medical oversight, causality assessment, and review [20-22].

The MM's activities are primarily conducted remotely. In rare circumstances, such as the occurrence of unexpected participant deaths at a phase 1 site, the MM may be requested to visit the site to assist with an audit and help determine the events and underlying causes. Moreover, having an MM bolsters readiness for inspections and audits by demonstrating a robust pharmacovigilance infrastructure. The MM’s documented reviews, queries, and recommendations form part of the trial’s safety file, which is a critical element in regulatory submissions and approval decisions [23].

Conclusion

The MM serves as a linchpin in modern clinical trial safety oversight, especially for small and emerging life sciences firms. Engaging a qualified MM improves safety signal detection, enhances protocol feasibility, and ensures regulatory alignment. As novel technologies continue to enter clinical development, the MM’s function becomes increasingly indispensable. Independent medical monitoring is not a formality; it is a strategic pillar of credible, compliant, and ethical drug and device development. The MM provides real-time clinical insight that supports early decision-making and protects patient welfare. By bridging scientific, clinical, and operational domains, the MM contributes directly to trial success, data integrity, and long-term public trust.
